# Short-term response might influence the treatment-related benefit of adjuvant chemotherapy after concurrent chemoradiotherapy for esophageal squamous cell carcinoma patients

**DOI:** 10.1186/s13014-021-01921-3

**Published:** 2021-10-02

**Authors:** Ao Liu, Yalin Wang, Xin Wang, Liqiong Zhu, Yu Nie, Minghuan Li

**Affiliations:** 1 Cheeloo College of Medicine, Shandong University, Jinan, China; 2 Department of Radiation Oncology, Qilu Hospital, Cheeloo College of Medicine, Shandong University, Jinan, China; 3 Department of Radiation Oncology, Shandong Cancer Hospital, Cheeloo College of Medicine, Shandong University, Jinan, China; 4 Department of Radiation Oncology, Shandong Cancer Hospital and Institute, Jinan, Shandong China; 5 Shandong First Medical University and Shandong Academy of Medical Sciences, Jinan, China

**Keywords:** Esophageal cancer, Radiotherapy, Adjuvant chemotherapy, Prediction

## Abstract

**Background:**

Whether adjuvant chemotherapy (AC) after concurrent chemoradiotherapy (CCRT) could provide benefit to esophageal squamous cell carcinoma (ESCC) patients is controversial. Therefore, we decided to investigate the potential benefit of AC after CCRT for ESCC and to identify biomarkers predictive of a clinical benefit.

**Methods:**

We retrospectively analysed the clinical data of ESCC patients with clinical stage II–IVa who underwent CCRT. Then, we compared patients who received CCRT and AC (CCRT + AC group) with those who received CCRT alone (CCRT group). Propensity score analysis, subgroup analysis and an additional Cox regression model were conducted to analyse the predictive factors. The overall survival (OS) and progression-free survival (PFS) rates were taken as the endpoints.

**Results:**

From January 2013 to December 2017, 244 patients were recruited (n = 131 for CCRT + AC; n = 113 for CCRT alone) for the analysis. After propensity score matching was performed (1:1 and 99 patients for each group) with consideration of the basic clinical characteristics, no significant differences were found in OS (HR = 1.024; 95% CI 0.737–1.423; *P* = 0.886) or PFS (HR = 0.809; 95% CI 0.582–1.126; *P* = 0.197) between the two groups. The good short-term response subgroup showed a better PFS and favoured CCRT + AC treatment (HR = 0.542; 95% CI 0.336–0.876; *P* = 0.008), the independent predictive role of which was confirmed in additional multivariate Cox regression analysis.

**Conclusions:**

Although AC did not significantly improve PFS and OS for all ESCC patients after CCRT, the short-term response to CCRT might help identify a subgroup that will benefit, which needs further prospective research to confirm.

## Introduction

In Asian countries, esophageal squamous cell carcinoma (ESCC) is the main pathological type of esophageal cancer and it ranks as the seventh most common cancer globally [1]. Because of the nonspecificity of early symptoms, most ESCC patients are diagnosed in the progression stage and lose the opportunity for radical surgery [2]. As a result, definitive concurrent chemoradiation (CCRT) plays an important role in the treatment of locally advanced ESCC patients who are not suitable for or unwilling to accept surgery [3]. However, the effect of CCRT is not satisfactory, with a 5-year survival rate of only approximately 30% [4]. Therefore, many efforts have been made to improve the survival outcomes of these patients through improvements of the radiotherapy technology and chemotherapy regimens, but the benefit is limited [5, 6].

Adjuvant chemotherapy (AC) is a treatment option that is intended to improve cancer control and has shown efficacy in many cancers [7, 8]. Although the RTOG 85-01 and PRODIGE-5 trials both took AC into account during their treatment design, the current guidelines do not recommend AC as a standard treatment [9, 10]. The role of AC after CCRT has not been explored in randomized controlled clinical trials, and the retrospective studies have yielded mixed results [11]. Some researchers found that AC could not increase survival benefits, while others showed that AC could improve survival [12, 13]. In addition, the different recommendations for AC may indicate that these patients need individualized treatment, and subgroups who might benefit need to be identified.

In such circumstances, we conducted retrospective research to analyse the role of AC in patients who underwent CCRT and identified subgroups that could benefit from AC to guide personalized treatment planning.

## Methods

### Study population

The records of esophageal squamous cell carcinoma patients with clinical stage II–IVa who underwent CCRT for initial therapy at Shandong Cancer Hospital between January 2013 and December 2017 were retrospectively reviewed.

### Ethics statement

Our retrospective study abided by the rules of medical ethics, and the Institutional Review Board (IRB) of Shandong Cancer Hospital approved this study. The number for the ethical statement was SDTHEC201902002. All patients were informed before treatment, agreed to receive concurrent CCRT and signed informed consent forms. We protected patient privacy and excluded patient identification information from our analysis.

### Inclusion and exclusion criteria

ESCC patients who met the following criteria were enrolled: (1) the patients were diagnosed by endoscopy combined with pathological biopsy-proven squamous cell carcinoma; (2) the clinical staging for each patient was defined according to the American Joint Committee on Cancer system (8th edition) for ESCC patients and clinically diagnosed with local advanced disease (stage II–IVa); (3) underwent definitive radiotherapy (dose ≥ 50.4 Gy, 1.8–2 Gy/fraction, three-dimensional conformal radiotherapy technology) with concurrent TP or PF doublet chemotherapy. (P indicates a type of platinum drug such as cisplatin, carboplatin or oxaliplatin, F indicates a fluoropyrimidine such as 5-fluorouracil or capecitabine, and T indicates a taxane such as paclitaxel or docetaxel) followed with or without AC; (4) patients who were in the 18–75 age range and whose Eastern Cooperative Oncology Group (ECOG) performance status (PS) score was no more than 2; and (5) patients who did not undergo salvage surgery during the follow-up for therapy response and survival evaluation.

The exclusion criteria were as follows: a prior treatment history, complications with other cancers, non-squamous cell carcinoma, clinical stage IVb, other chemotherapy regimens, non-definite radiotherapy (dose < 50.4 Gy) and patients who were lost to follow-up.

### Radiotherapy

Radiotherapy was delivered using 6 MV linear accelerators with normal fractionation (total ≥ 50.4 Gy, each fraction dose in the 1.8–2.0 Gy range, five days for therapy and two days for intervals every week). All patients underwent RT with three-dimensional conformal radiotherapy. The gross target volume (GTV) included lesions that could be confirmed by upper gastrointestinal radiography, endoscopy or contrast-enhanced computed tomography (CT). Clinical target volume (CTV): Elective lymph node irradiation (ENI) or involvement field irradiation (IFI) may be considered for recipients, and both target delineation methods are acceptable.

IFI: the front, back, left, right, up and down directions of the borders were 5–6 mm beyond the GTV (this was then adjusted to include anatomical barriers).

ENI: in addition to the primary lesion and metastatic lymph node area, the lymph node regions that were considered to be at high-risk for metastasis with regard to tumour location should also receive radiation. The planning target volume (PTV) was defined as the region created from the CTV and its isotropic expansion for 5 mm.

### Chemotherapy

CCRT was defined as the start of chemotherapy at the same time as radiotherapy (within a range of 2 weeks earlier up to 1 week later). AC was defined as additional chemotherapy administered after CCRT under the circumstance that there was no sign of progression of the cancer. The initiation of AC was approximately 2–6 weeks after CCRT. The chemotherapy schedule was TP or PF doublet chemotherapy. (P refers to a platinum drug such as cisplatin, carboplatin or oxaliplatin, F refers to a fluoropyrimidine such as 5-fluorouracil or capecitabine, and T refers to a taxane such as paclitaxel or docetaxel). Every concurrent chemotherapy cycle was 3 weeks, and two cycles were given, while AC was repeated every 3 weeks for 3 and 6 cycles.

### Evaluation and follow-up

Every enrolled patient had full basic clinical information collected, such as sex, age, ECOG PS score, upper gastrointestinal radiography, endoscopy and enhanced CT results, before undergoing primary therapy. We defined the short-term tumour response as the response estimation, which was evaluated just one month after finish CCRT through a follow-up physical examination, upper gastrointestinal radiography, endoscopy, and enhanced CT. The recognized response evaluation criteria (Response Evaluation Criteria in Solid Tumours 1.1 (RECIST 1.1)) have categoried responses into complete remission (CR), partial remission (PR), stable disease (SD) and progressive disease (PD). The good short-term tumour response patients exhibited a CR or PR, and a poor short-term tumour response manifested as SD or PD.

Furthermore, the toxicities associated with treatment were evaluated every cycle, which followed the Common Terminology Criteria for Adverse Events 3.0 (CTCAE 3.0).

The surveillance for these ESCC patients included upper gastrointestinal radiography, endoscopy and contrast-enhanced computed tomography (CT) to estimate the change of disease and associated blood examinations to assess their general condition. Surveillance was conducted at a frequency of once every 3 months in the first 2 years and once every 3 months for the following 5 years until death.

### Statistical analysis

Overall survival (OS) was recognized as the duration between the initiation of therapy and death from any cause or the time of lost to follow-up. Progression-free survival (PFS) was measured from the beginning of primary therapy to recurrence or death. The OS and PFS of different groups of patients were plotted by the Kaplan–Meier method, and differences were analysed by means of the log-rank test. We compared the baseline clinical characteristics between the two groups via Pearson’s chi-squared test.

With regard to the influence of potential selection bias and confounding factors, we conducted propensity-score matching (PSM) to minimize any effect between different groups. Participants (CCRT + AC and the CCRT alone cohort) were 1:1 matched using the nearest neighbour algorithm (the caliper was calculated as 0.2 times of the standard deviation of the logit of the propensity score, which was random matching order and no replacement was done). Predictors were identified by subgroup analysis. A new prediction factor screening method used in this research as a supplementary method defined the OS or PFS benefit of AC as exceeding the median OS or PFS of the CCRT alone group patients, which has been trained and validated in previous studies [14, 15], scored as 1 for benefit and 0 for no benefit. Then, univariate and multivariate logistic analyses were utilized to screen for potential independent predictive factors. Statistical analysis and related graphs were conducted and plotted by means of SPSS 24.0 (IBM Corporation, Armonk, NY, USA), GraphPad Prim 9 and R version 4.0.3 software (http://www.r-project.org/). All P values were calculated as two-sided, and values less than 0.05 indicated a significant difference.

## Results

### Screening process

**Fig. 1:**
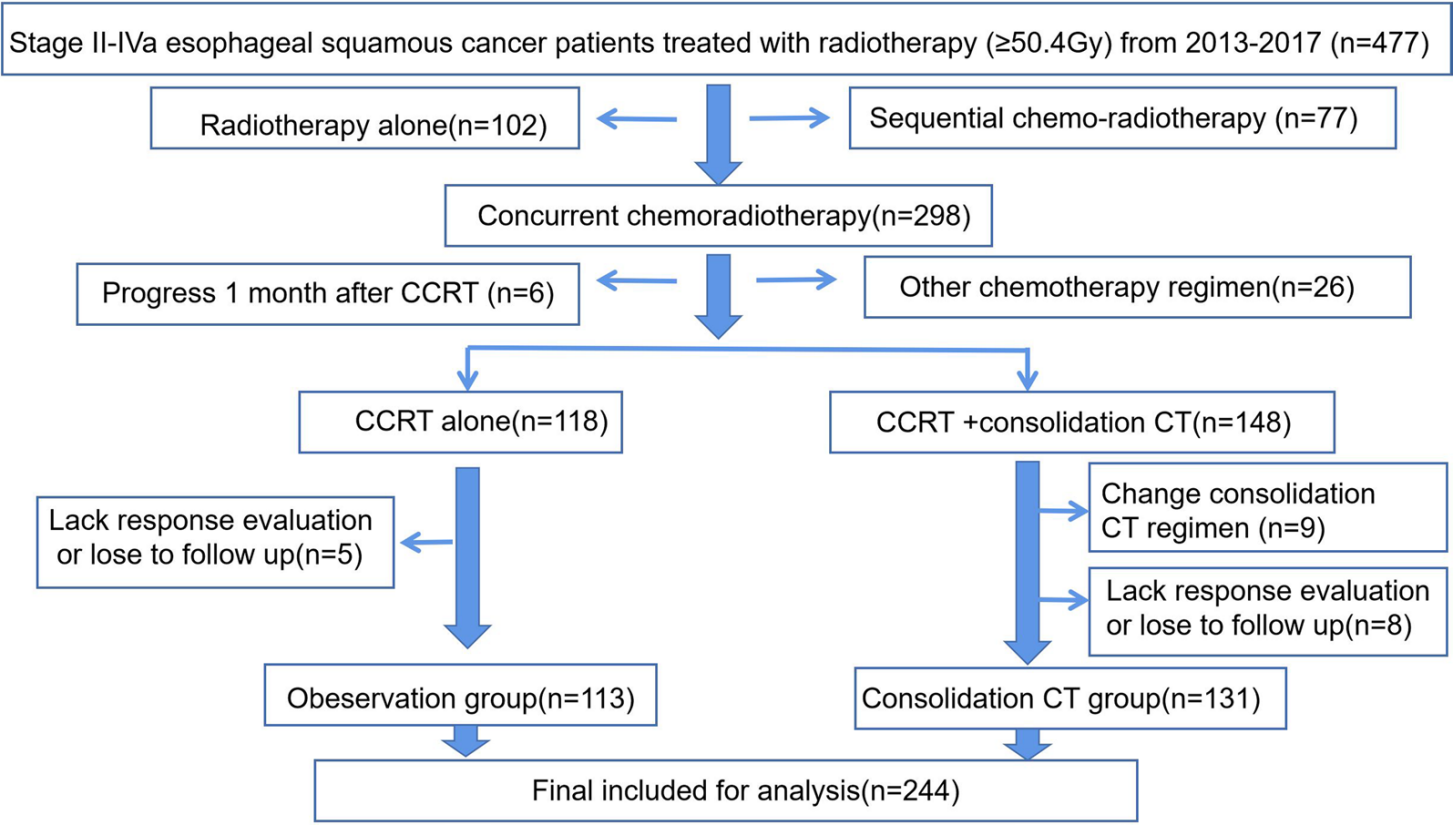
The screening process of clinical stage II–IVa esophageal squamous cell cancer patients treated with radiotherapy

Four hundred and seventy-seven consecutive ESCC patients who were clinical staged II–IVa and underwent radiotherapy (> 50.4 Gy) as initial therapy were screened first. Then, the following patients were excluded due to not meeting the research requirements: 102 patients only underwent radiotherapy alone, 77 underwent sequential but not concurrent chemotherapy and radiotherapy, 26 did not undergo a doublet chemotherapeutic regimen of PF/TP, and 6 patients experienced progression 1 month after CCRT and then received second-line treatment. Five patients in the CCRT alone group lacked response evaluation or were lost to follow-up. Eight patients in the CCRT + AC group lacked response evaluation or were lost to follow-up. Nine patients received adjuvant chemotherapy regimens that were different from their concurrent CCRT regimens (Fig. 1).

### Patient characteristics

After the screening, 244 patients were included in the analysis, including 201 male patients (82.4%), median age 60 (range 38 to 81) at the time of first treatment. A total of 137 (56.1%) patients had an ECOG score of 0, 164 (67.2%) patients had clinical stage II–IVa diseases, 131 (53.7%) patients received a chemotherapy regimen of PF, and 120 (49.2%) patients received radiotherapy using the IFI target delineation method. The median radiation dose was 59.4 Gy (range 50.4–66 Gy). A total of 135 (55.3%) patients achieved good short-term responses of CR or PR 1 month after finishing CCRT. A total of 113 (46.3%) patients underwent CCRT alone, and 131 (53.7%) patients received CCRT + AC. The baseline characteristics are summarized in Table 1. Before matching, more patients were clinical stage III (61.6% vs. 45.1%) in the CCRT + AC group. After propensity score matching, the CCRT + AC group and CCRT alone group included 99 patients each, and their baseline clinical features were well balanced and showed no significant differences (*P* > 0.05) (Table 1).

**Table 1:** Patient demographic and clinical characteristics before and after PSM

	Before PSM				After PSM			
	CCRT	CCRT + AC	X2	*p*	CCRT	CCRT + AC	X2	*p*
Sex			1.081	0.298	99	99	0.033	0.855
Male	90	111			81	80		
Female	23	20			18	19		
Age			1.217	0.270			0.342	0.559
< 60	43	59			40	36		
≥ 60	70	72			59	63		
ECOG scoring			0.401	0.527			0.082	0.775
0	61	76			54	56		
1–2	52	55			45	43		
Tumor location			0.489	0.921			0.416	0.937
Cervical	9	10			9	10		
Up	53	62			45	48		
Middle	35	44			32	28		
Down	16	15			13	13		
Clinical stage			6.334	0.042			0.617	0.735
II	19	14			16	13		
III	51	80			48	53		
IVA	43	37			35	33		
T stage			1.576	0.455			0.807	0.668
2	21	17			18	15		
3	69	83			62	68		
4	23	31			19	16		
N stage			3.062	0.382			0.527	0.913
0	18	27			17	15		
1	50	65			45	50		
2	39	35			34	31		
3	6	4			3	3		
MacroType			1.044	0.791			1.275	0.735
Fungating	27	26			23	24		
Ulcer	35	41			34	28		
Medulla	33	45			25	31		
Constrictive	18	19			17	16		
CT regimen			1.885	0.170			0.081	0.775
PF	66	65			53	55		
TP	47	66			46	44		
Radiation dose			0.118	0.731			0.028	0.867
< 59.4 Gy	28	30			23	24		
≥ 59.4 Gy	85	101			76	75		
Target delineation			1.942	0.163			0.081	0.776
IFI	61	59			52	50		
ENI	52	72			47	49		
Short-term response			0.410	0.522			0.020	0.887
Good	65	70			53	54		
Poor	48	61			46	45		

*PSM* propensity score matching, *ECOG* Eastern Cooperative Oncology Group

### Survival outcome and failure pattern

The last follow-up was December 31, 2020, while the median time of surveillance was 32 months (range 3–94 months). The 1-, 3-, and 5-year PFS rates for the cohort before matching were 63.5%, 36.1%, and 23.2%, respectively. The 1-, 3-, and 5-year OS rates for the cohort before matching were 84.8%, 48.4%, and 17.2%, respectively. The median PFS was calculated to be 20.0 months, while the median OS was 32.5 months.

**Fig. 2:**
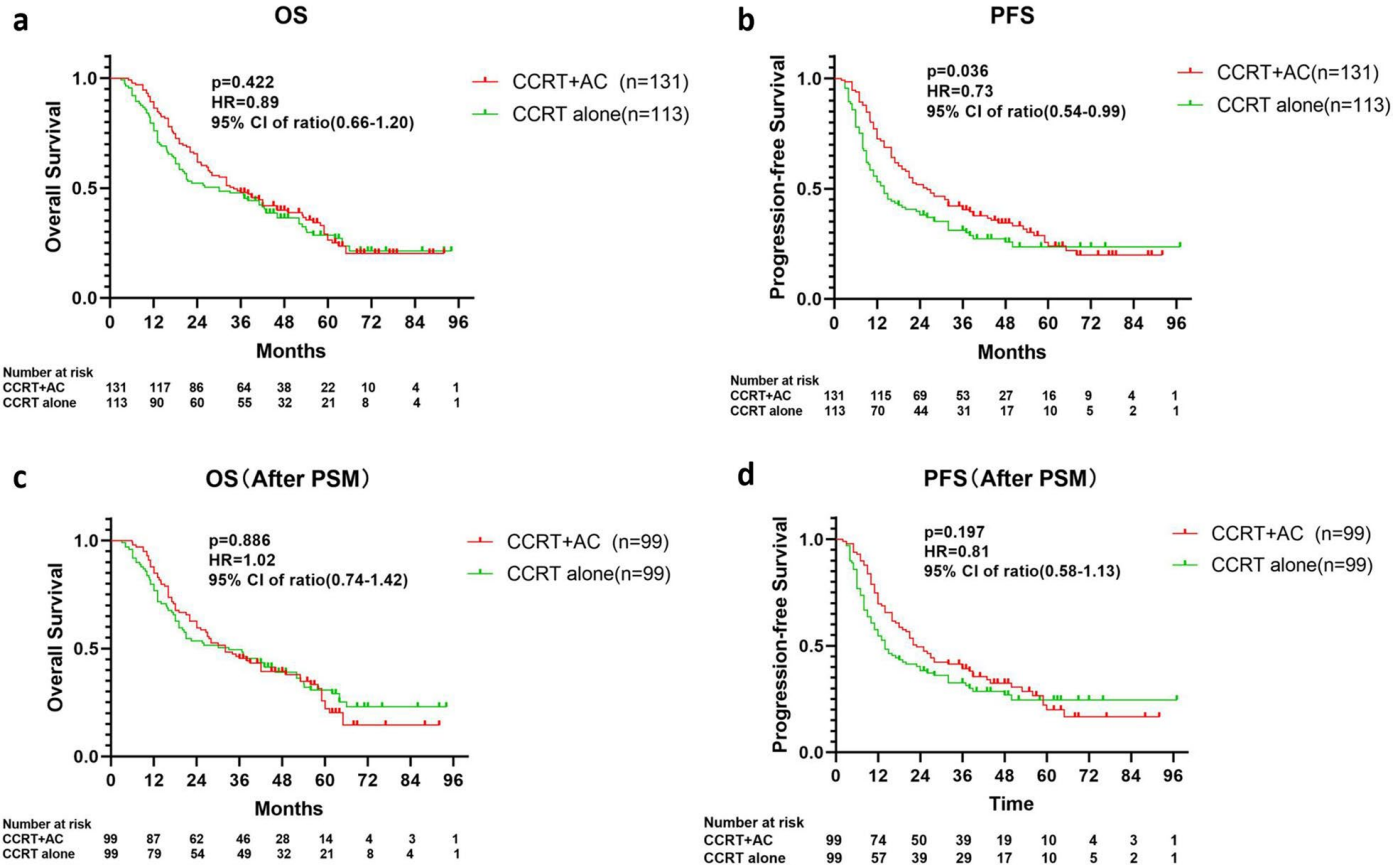
Overall survival and progression-free survival before (**a**, **b**) and after (**c**, **d**) PSM. Abbreviations: PSM, propensity score matching

The median OS in the CCRT alone group was 30.0 months, while that in the CCRT + AC group was 34.0 months, which was not significantly different (hazard ratio [HR] = 0.89 (95% CI 0.66–1.20), *P* = 0.422; Fig. 2a). However, the CCRT alone group (14.0 months) exhibited a significantly shorter median PFS than the CCRT + AC group (26.0 months) (HR = 0.73 (0.54–0.99), *P* = 0.036; Fig. 2b).

The 1-, 3-, and 5-year PFS rates for the cohort after PSM were 62.1%, 36.0%, and 21.9%, respectively. The 1-, 3-, and 5-year OS rates for the cohort after PSM were 80.8%, 47.0%, and 26.9%, respectively. The median PFS was calculated to be 19.0 months, while the median OS was 32.0 months.

In the matched population, the median OS in the CCRT alone group was 33.0 months, while that in the CCRT + AC group was 32.0 months, which was not significantly different (HR = 1.024 (0.74–1.42), P = 0.886; Fig. 2c). The CCRT alone group (14.0 months) exhibited a shorter median PFS than the CCRT + AC group (23.0 months), but the difference was not significant (HR = 0.81 (0.58–1.13), *P* = 0.197; Fig. 2d).

We also collected failure pattern data for these patients, and the results are shown in Table 2. The CCRT alone cohort exhibited a slightly higher rate of locoregional combined with distant failure than the CCRT + AC cohort before PSM (18.6% vs. 14.5%) and after PSM (16.2% vs. 19.2%). However, the difference was not statistically significant.

**Table 2:** Patterns of failure for patients who underwent AC before and after PSM

Pattern of failure, n(%)	Before PSM				After PSM			
	CCRT alone (n = 113)	CCRT + AC(n = 131)	χ^2^	*p*	CCRT alone (n = 99)	CCRT + AC (n = 99)	χ^2^	*p*
Locoregional alone	38 (33.6)	47 (35.9)	0.784	0.853	31 (31.3)	38 (38.4)	3.032	0.387
Locoregional and distant	21 (18.6)	19 (14.5)			20 (20.2)	12 (12.1)		
Distant alone	19 (16.8)	24 (18.3)			16 (16.2)	19 (19.2)		
No failure	35 (31.0)	41 (31.3)			32 (32.3)	30 (30.3)		

*AC* adjuvant chemotherapy, *PSM* propensity score matching

### Subgroup analysis

**Fig. 3:**
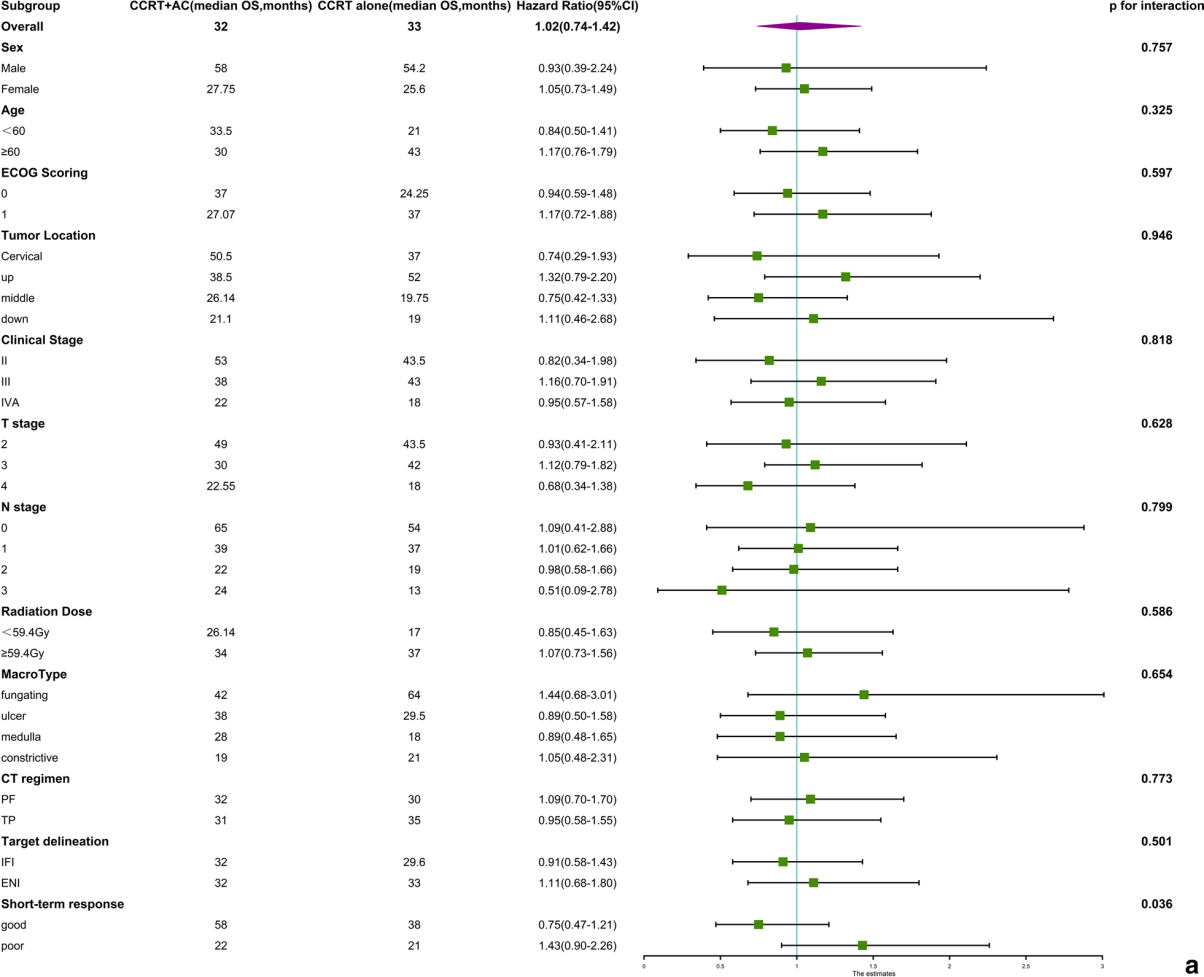

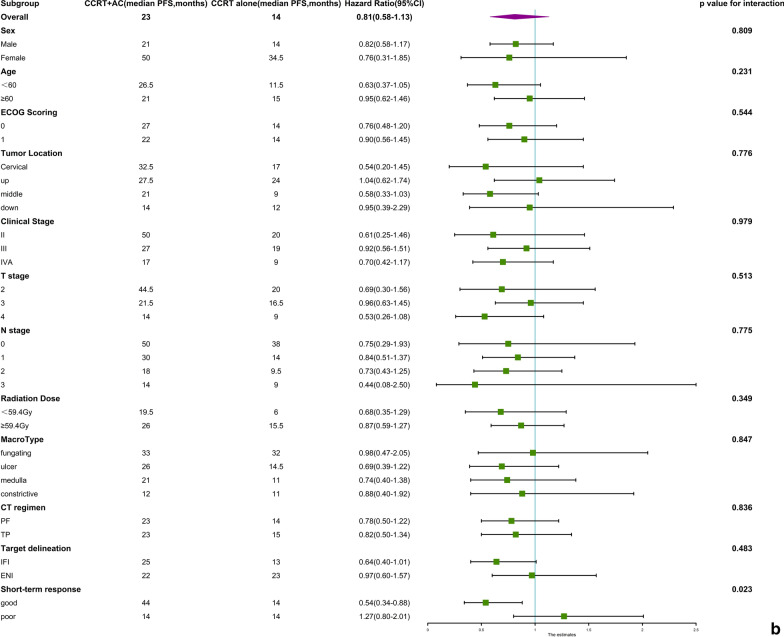
**a** Subgroup analysis for overall survival. **b** Subgroup analysis for progression-free survival

In the OS subgroup analysis, none of the basic clinical characteristics in the analysis showed a significant predictive effect. It is worth mentioning that the influence of the short-term response on survival was significant (p for interaction 0.036; Fig. 3a), although neither subgroup reached a significant difference.

In the PFS subgroup analysis, the influence of the short-term response on survival was significant (p for interaction 0.023). Only the good short-term response subgroup favoured adjuvant chemotherapy (CCRT + AC versus CCRT alone, HR = 0.54 (0.34–0.88), *P* = 0.008). The other basic clinical characteristics in the analysis did not show a significant predictive effect (Fig. 3b).

**Fig. 4:**
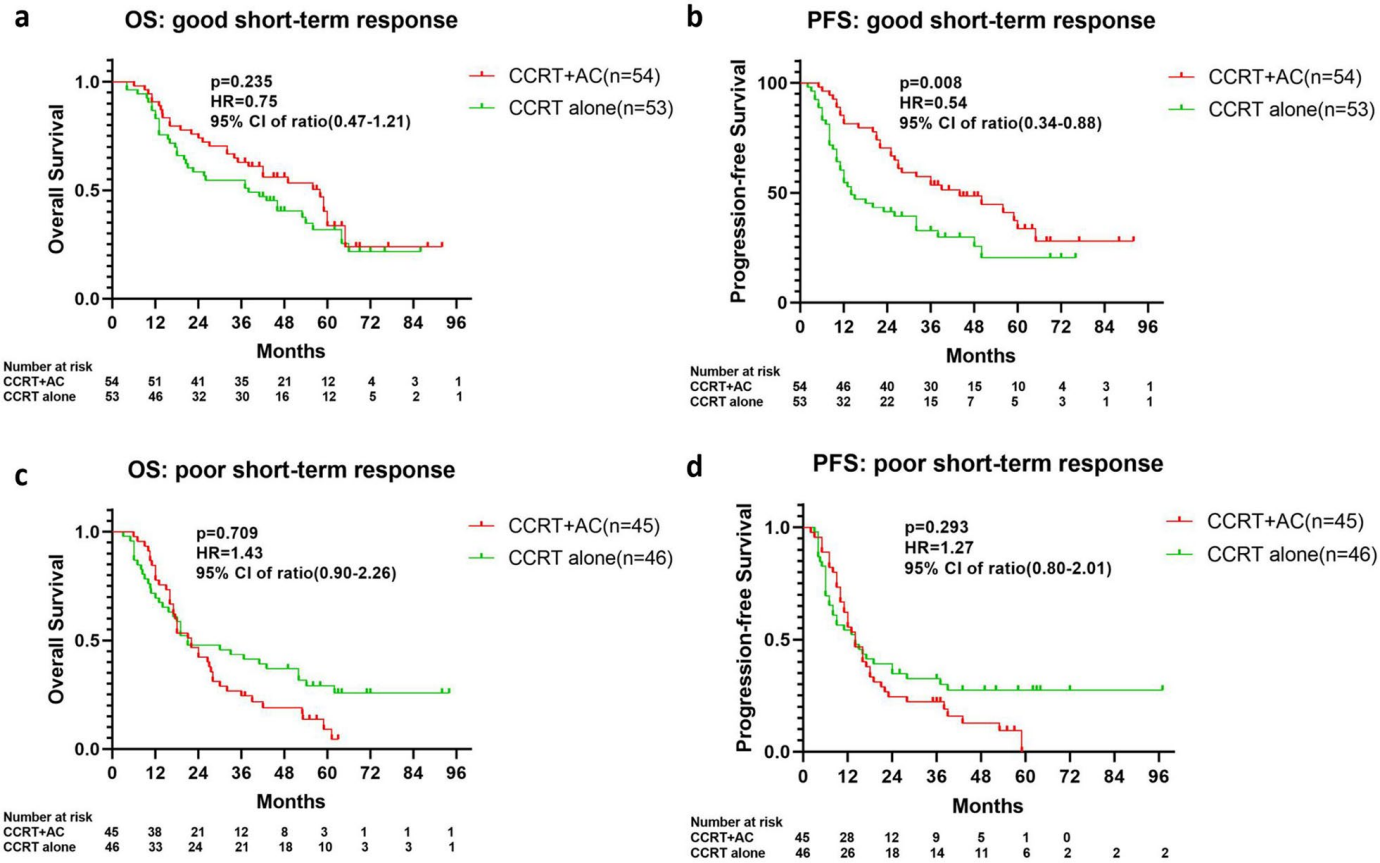
Overall survival and progression-free survival analysis for the good (**a**, **b**) and poor (**c**, **d**) short-term response subgroups

Details of the survival outcomes of the short-term response group were calculated. For patients with a good short-term response, the CCRT alone cohort did not show a significant OS difference compared with the CCRT + AC cohort (median, 38.0 vs 58.0 months, (HR = 0.75 (0.47–1.21), *P* = 0.235; Fig. 4a). However, the CCRT alone cohort exhibited shorter PFS than the CCRT + AC cohort (median, 14.0 vs. 44.0 months, HR = 0.54 (0.34–0.88), *P* = 0.008; Fig. 4b).

For those patients with a poor short-term response, the CCRT alone cohort did not show significant OS (median, 21.0 vs 22.0 months, (HR = 1.43(0.90–2.26), *P* = 0.709; Fig. 4c) or PFS (median, 14.0 vs. 14.0 months, HR = 1.27(0.80–2.01), *P* = 0.293; Fig. 4d) differences compared with the CCRT + AC cohort.

### AC cycles analysis

**Fig. 5:**
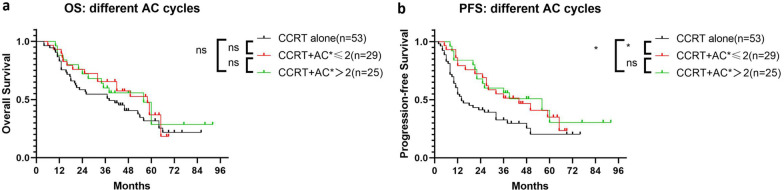
Overall survival and progression-free survival analysis for patients with different numbers of AC cycles. Abbreviations: AC, adjuvant chemotherapy

The median number of AC cycles was 2 and it ranged from 1 to 4. Considering the potential survival benefit of adjuvant chemotherapy for the good short-term response subgroup, we further analysed the influence of AC cycles. Compared with the patients who received two cycles of AC, receiving more than two cycles of AC chemotherapy did not significantly improve PFS or OS (Fig. 5a, b).

### AC benefit analysis

After PSM, the median value for OS was 33.0 months and that for PFS was 14.0 months in the CCRT alone cohort. As a result, the OS or PFS benefit of AC was defined as exceeding the median OS or PFS of the CCRT alone group patients. Coding 1 for benefit and 0 for no benefit, all survival times of the patients could be transferred to categorical variables based on the AC benefit. Then, univariate logistics analysis showed that sex (male or female), clinical stage (stage II–III or/stage IV), N stage (stage N0-1 or N2-3), short-term response (good or poor) were potential predictive factors for OS, while sex (male or female), clinical stage (stage II–III or stage IV), T stage (stage T2-3 or stage T4), short-term response (good/poor) were potential predictive factors for PFS. Further multivariate analysis showed that a short-term response (good/poor) was a significant influencing factor for OS, and a short-term response (good/poor) and macrotype (fungating + ulcer/medulla + constrictive) were potential influencing factors for PFS. Thus, the influence of a short-term response on the outcome of AC benefit is further proven (Table 3).

**Table 3:** Univariate and multivariate analyses for prognostic factors of AC benefit through the transformation model

Prognostic factors	Univariate analyses				Multivariate analyses			
	OS		PFS		OS		PFS	
	*P*	HR (95.0% CI)	*P*	HR (95.0% CI)	*P*	HR (95.0% CI)	*P*	HR(95.0% CI)
Gender Male/female	0.059	2.786 (0.962–8.068)	0.069	3.374 (0.908–12.538)	0.251	2.071 (0.598–7.174)	0.204	2.513 (0.606–10.421)
Age < 60/ ≥ 60	0.820	0.909 (0.401–2.063)	0.873	0.932 (0.392–2.213)				
ECOG Scoring 0/1–2	0.249	0.624 (0.280–1.391)	0.743	1.151 (0.497–2.666)				
Tumor location Up/down	0.115	0.520 (0.231–1.173)	0.212	0.585 (0.253–1.356)				
Clinical stage II–III/IV	0.001	0.208 (0.082–0.531)	0.102	0.485 (0.204–1.156)	1.000			
T stage T2-3/T4	0.139	0.423 (0.135–1.324)	0.050	0.335 (0.112–1.001)			0.064	0.304 (0.086–1.070)
N stage N0-1/N2-3	0.002	0.240 (0.097–0.596)	0.141	0.523 (0.221–1.240)	1.000			
Radiation dose < 59.4 Gy/ ≥ 59.4 Gy	0.444	1.438 (0.568–3.641)	0.177	1.913 (0.746–4.903)				
MacroType Fungating + Ulcer/Medulla + Constrictive	0.129	0.538 (0.242–1.197)	0.004	0.280 (0.116–0.673)			0.005	0.246 (0.092–0.661)
CT Regimen PF/TP	0.893	0.947 (0.428–2.093)	0.962	1.020 (0.443–2.352)				
Target Delineation ENI/IFI	0.922	1.040 (0.473–2.288)	0.726	1.160 (0.506–2.662)				
Short-term response Good/poor	< 0.001	5.500 (2.305–13.126)	< 0.001	5.029 (2.039–12.399)	< 0.001	5.969 (2.258–15.776)	0.003	4.395 (1.650–11.710)

## Discussion

Although CCRT is now recognized as the standard primary therapy strategy for ESCC patients who refuse or are ineligible for surgery, it still has a high recurrence rate and an unsatisfactory survival outcome [16]. Many efforts have been made with the hope of improving CCRT regimens, such as optimizing radiotherapy (innovation technology, modified dose and target delineations) and exploring chemotherapy regimens, but they have achieved little benefit [5, 6]. As a result, we conducted this research to investigate the efficacy of AC after CCRT for ESCC to identify biomarkers predictive of a clinical benefit from AC.

Although the current National Comprehensive Cancer Network guidelines do not recommend adjuvant chemotherapy as a standard treatment regimen for locally advanced ESCC patients, many large-scale clinical trials have included AC as an optional scheme [9, 10]. In addition, many oncologists consider consolidation chemotherapy for ESCC patients after CCRT to improve survival outcomes. In addition, no RCTs have been conducted to explore the efficacy of AC after CCRT. Several retrospective studies have focused on this issue, but the results are confusing. Koh et al. [17], Wu et al. [13] and Zhang et al. [18] found that AC could improve OS, while other studies did not show a significant survival benefit from AC for patients who underwent CCRT [12, 19–21].The potential benefit of AC for PFS has been confirmed by Koh et al. [17] and Chen et al. [20] but not those of Wu et al. [13] and Chen et al. [12]. The inclusion of patients who underwent induction chemotherapy in the research of Koh et al. [17] and patients of stage I in the research of Wu et al. [13] and Zhang et al. [18] might have an effect in benefit evaluation of AC. The baseline clinical features of patients who underwent CCRT and AC and CCRT alone did not undergo PSM in the research of Chen et al. [20] also increased the confusion for AC benefit for whole ESCC patients without selection. We found that AC did not significantly improve OS for ESCC patients after CCRT and AC could increase PFS before PSM but it lost this advantage after PSM. Due to the retrospective nature of all of the studies and the heterogeneity between different areas and medical centres, the potential additional chemotherapy-related benefit may favour AC, while the increasing possibility of adverse reactions could attenuate the benefit. We thought the benefit of AC might be controversial, but patients suitable for this therapy need careful selection, and an individual approach to treatment is important.

Short-term response was confirmed as a significant predictive factor for AC benefit in our studies. Since the short-term response is routinely evaluated in almost every medical centre, its relevance to the potential benefit may be very useful. Subgroup analysis and the new parameter-transformed methods have been tested for rationality in previous studies [14, 15]. A possible explanation for the benefit is that sensitivity to chemotherapy may reflect the possible benefit, and the rapid decline in tumour burden may contribute to subsequent tumour control. Adding benefit for patients with good response may be easier than achieving qualitative change for those with poor response. Thus the short-term response evaluation should be included in the future clinical trail design for different groups due to its specific role of benefit prediction. More biomarkers which could reflect short-term response need more attentions such as the tumor markers from liquid biopsy and functional imaging. The potential benefit for those patients with good short-term response should be confirmed in the future prospective researches. In addition, the exploration for patients with poor short-term response evaluation is urgent since the current regimens did not show satisfactory effect.

We also found that an additional two cycles were sufficient and that more treatment may not lead to more benefit, which provides evidence to support the two-cycle AC regimen of past large-scale trials. In addition, 2 cycles of chemotherapy for AC and 2 cycles of concurrent CRT amount to 4 cycles of chemotherapy, similar to most chemotherapy regimens that are used regularly. Chemotherapy plays an important role in CCRT as radiation sensitizer and anti-tumor factor due to its intrisic pharmacological basis which is closely related to the cancer sensitivity. Thus sensitivity evaluation is more important. Increase the number of chemotherapy cycles without selection may add more side effects than benefit. Therefore optimal cycle number should be identified. The published clinical studies did not specifically compared the effects of more than two cycles regimen with two cycles treatment. Our research showed that 2 cycles of AC should be considered for patients with a good short-term response to CCRT. However, due to the nature of retrospective studies, the conclusion requires further confirmation through preclinical and prospective clinical studies.

Other clinical factors were also investigated, but none were good predictors of a benefit. As the benefit of AC may be influenced by the tumour biological features, future research should focus on the relationship of genetic characteristics and the AC benefit. Molecular typing has been researched extensively since the publication of genetic differences between ESCC and EAC [22]. Similar to the chemotherapy risk score system for breast cancer and other cancers, a method of selecting patients who can benefit from AC should also be created [23]. In addition, radiomics and machine-learning models have been proven to provide valuable information for clinical decision-making, which could also be applied to this topic [24]. Furthermore, the different immune infiltration features may affect the treatment outcomes, which may also be related to the AC benefit [25, 26].

It should be noted that although AC could improve the outcome of patients with a good short-term response, those with a poor short-term response need a different approach to treatment. Immune checkpoint inhibitors after CCRT have shown remarkable survival benefits in NSCLC [27]. Immunotherapy, typified by ICIs, combined with new chemotherapy drugs and new targeted therapy may have a role and should be explored further for these patients [25].

Our research also has several limitations that should be kept in mind. Retrospective studies and limited population sizes may cause selection bias. Although PSM methods have been applied to minimize the effect of bias, large prospective studies are necessary to confirm our findings. The quality of life was not assessed due to the lack of certain medical records, and it may influence the decision about AC. In addition, our research mainly focused on TP and PF chemotherapy regimens, while other chemotherapy regimens were excluded to reduce heterogeneity in the analysis. However, both regimens are recommended by most guidelines and are commonly used. In addition, a certain number of stage IVa patients were included, which might be a potential difference from other studies but should not interfere with the conclusions. Therefore, large and prospective clinical investigations are urgently needed to further explore the potential benefit of AC and associated biomarkers.

## Conclusions

In conclusion, many oncologists consider consolidation chemotherapy for ESCC patients after CCRT to improve survival outcomes, but the efficacy of AC after CCRT is controversial. A good short-term response has been confirmed as a significant predictive factor for AC benefit in our study, which needs further exploration. More predictive biomarkers and models should be studied to help select the subpopulation most likely to benefit from AC.

## Data Availability

The datasets used and/or analyzed during the current study are available from the corresponding author on reasonable request.
